# Integrating morphological, anatomical, and physiological traits to explain elevational distributions in Himalayan steppe and alpine plants

**DOI:** 10.1111/jipb.13971

**Published:** 2025-07-15

**Authors:** Jan Binter, Martin Macek, Jiri Dolezal

**Affiliations:** ^1^ Institute of Botany of the Czech Academy of Sciences Průhonice 252 43 Czech Republic; ^2^ Department of Experimental Plant Biology, Faculty of Science Charles University Prague 128 43 Czech Republic; ^3^ Department of Experimental Plant Biology, Faculty of Science University of South Bohemia České Budějovice 370 05 Czech Republic; ^4^ Department of Botany, Faculty of Science University of South Bohemia České Budějovice 370 05 Czech Republic

**Keywords:** alpine plants, climate change, elevational gradient, mountain ecosystems, plant functional traits, species distribution

## Abstract

Understanding plant adaptive strategies that determine species distributions and ecological optima is crucial for predicting responses to global change drivers. While functional traits provide mechanistic insights into distribution patterns, the specific trait syndromes that best predict elevational optima, particularly in less‐studied regions such as the Himalayas, remain unclear. This study employs a novel hierarchical framework integrating morphological, anatomical, and physiological traits to explain elevational distributions among 310 plant species across a 3,500‐m gradient (2,650–6,150 m). We analyzed 95,000 floristic records collected from 4,062 localities spanning 80,000 km^2^ in Ladakh, NW Himalayas, India, to define elevational optima and link them with 17 functional traits from over 7,800 individuals. We assessed the roles of moisture and cold limitations on trait–optima relationships by comparing two contrasting habitats (dry steppe and wetter, colder alpine). The predictive power of functional traits was more pronounced in the alpine species facing more extreme abiotic stress than the steppe species. Our results indicate that conservative life history strategies strongly predict elevational optima in alpine areas, while drought avoidance and competitive dominance are key in steppe habitats. Trait syndromes combining short stature, compact growth forms, enhanced storage tissues, and features promoting water‐use efficiency (δ^13^C), freezing resistance (fructan levels), and nutrient retention (high root nitrogen and leaf phosphorus) explained 61% of the variation in alpine species' optima. Conversely, lifespan and clonal propagation determined the optima of steppe species at lower elevations. The study emphasizes the importance of functional trait combinations in determining elevational optima, highlighting that alpine species prioritize resource conservation and stress tolerance, while steppe species focus on competitive growth strategies. This multi‐trait approach contrasts with previous research focusing on single trait–elevation relationships, providing novel insights into the diverse mechanisms shaping elevational distributions and offering valuable predictive power for assessing vegetation responses to future climate change.

## Graphical abstract

Functional trait syndromes predict plant elevational optima across 310 Himalayan species. In alpine zones, small stature, storage tissue, and high δ^13^C/fructan drive cold adaptation; in steppe, height and clonality reflect drought and competition. Trait‐based models explain 61% of alpine optima, offering predictive power under climate change.

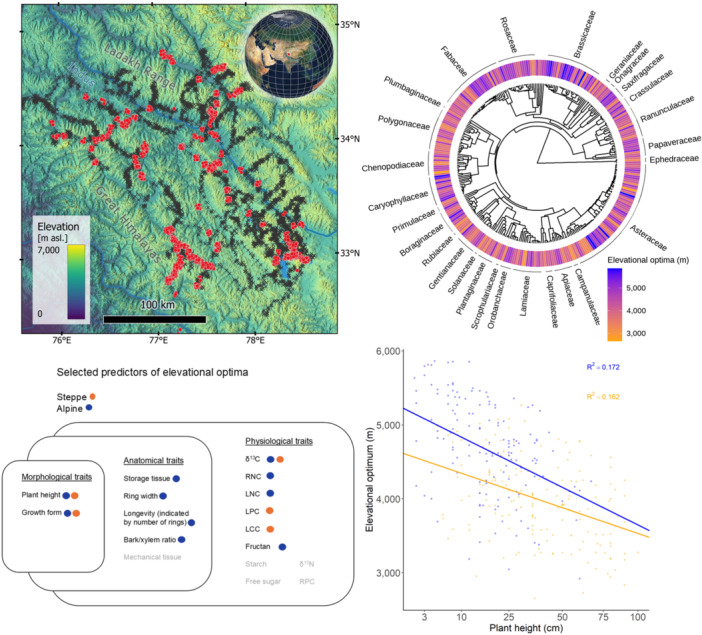

## INTRODUCTION

Understanding the mechanisms that shape species distributions along environmental gradients is essential for predicting their responses to global change drivers (Whittaker, 1967; Steinbauer et al., 2018; Liu et al., 2022). Plant functional traits provide valuable mechanistic insights into interspecific differences in elevational optima and range limits (McGill et al., 2006; Maharjan et al., 2021). Competitive traits often explain species distributions in favorable conditions, while stress‐tolerant traits dominate in harsher environments (Grime, 1974; Garnier et al., 2015; Körner, 2021). However, the specific combinations of traits that best predict elevational optima across extensive geographical gradients, particularly in vast mountain ranges like the Himalayas with steep elevational gradients spanning thousands of metres, remain unclear (Stahl et al., 2014; Dolezal et al., 2016). Elucidating how contrasting traits interact to define species' ecological niches is vital for forecasting plant responses to climate change (Madsen‐Hepp et al., 2023; Henn et al., 2024).

Mountain ecosystems are characterized by steep environmental gradients, where conditions change rapidly with elevation (Körner, 2021). These gradients significantly influence plant distribution patterns (Dvorský et al., 2017; Macek et al., 2021), driven by varying levels of plant adaptations (Westoby et al., 2002), which are reflected in interspecific differences in morphological, anatomical, and physiological traits (Maharjan et al., 2021). While numerous studies have explored the relationship between individual traits and elevation (e.g., Midolo et al., 2019; Zhang et al., 2019; Chlumská et al., 2022; Chondol et al., 2024), fewer have examined how trait combinations collectively influence species distributions across broader gradients (e.g., Dvorský et al., 2015; Sigdel et al., 2023; Henn et al., 2024). Such integrative approaches are critical for uncovering broader ecological mechanisms and improving predictions of plant responses to environmental change (Maharjan et al., 2021).

Increasing evidence highlights the importance of trait syndromes in determining species distributions (Hulshof et al., 2013; Tang et al., 2022), including species' elevational optima, which correspond to the elevations where species are most abundant and best adapted to limiting factors such as cold, drought, disturbances, nutrient deficiencies or competition (Grime, 1974). At lower elevations, where resources such as nutrients are more abundant, intense competition favors traits like rapid growth, larger size, and efficient resource acquisition (Garnier et al., 2015). Conversely, higher elevations are characterized by harsher abiotic conditions, including low temperatures, high winds, frost, and nutrient‐poor soils. Here, survival relies more on conservative traits that enhance tolerance to abiotic stress and resilience to disturbances (Körner, 2021). These contrasting pressures along the elevational gradient underline the dual role of competitive resistance at lower elevations and stress tolerance at higher elevations in shaping species distribution (Grime, 1974; Ratier‐Backes et al., 2023).

This study employs a novel hierarchical framework to examine how three interrelated trait categories—morphological, anatomical, and physiological—account for interspecific differences in elevational optima among over 300 dicot herb and shrub species across a 2,650–6,150 m elevation gradient in the western Himalayas. These trait groups represent hierarchical levels of adaptation: morphology influences plant–environment interactions (e.g., size and growth form, Doležal et al., 2024), anatomy governs key physiological processes such as water transport and freeze resistance (Venturas et al., 2017; Schweingruber et al., 2020), and physiology reflects strategies for resource acquisition and stress tolerance (Abeynayake et al., 2015; Hiltbrunner et al., 2021). For instance, at higher elevations, smaller plants reduce exposure to abiotic stresses (Liancourt et al., 2020), while anatomical adaptations, such as increased parenchyma tissue, enhance storage capacity and hydraulic repair (Dolezal et al., 2019a). Physiological traits, including water‐use efficiency (WUE) and non‐structural carbohydrate (NSC) content, further support survival in cold, resource‐scarce environments (Vijn and Smeekens, 1999; MacNeill et al., 2017).

By integrating these groups of traits, we aim to elucidate the mechanisms linking functional traits to plant distributions along elevational gradients (Ratier‐Backes et al., 2023). This study utilizes 95,000 floristic records from 4,062 localities covering 80,000 km^2^ in Ladakh, Trans‐Himalayas, India, to define species elevational optima among 310 plant species and link them with 17 functional traits from over 7,800 individuals. Our sequential analysis, starting with morphological traits and followed by anatomical and physiological characteristics, enabled us to account for the interconnectedness among traits and clarify their collective influence on species' elevational distributions. This integrative approach offers novel insights into the trait syndromes driving plant distributions along a 3,500‐m gradient (2,650–6,150 m) in underexplored western Himalayan mountain ecosystems.

The Ladakh region, situated in the rain shadow of the Great Himalayan Range, experiences overall aridity (50–150 mm of precipitation) and is predominantly treeless. Dry semi‐deserts and steppes, with extensive alpine grasslands along glacial rivers and lakes, characterize it. We thus investigated the relationships between species elevational optima and plant adaptive traits separately for the two main contrasting habitats in the cold‐arid Ladakh region of the western Himalayas (Dvorský et al., 2011, 2015, 2017): the drier, lower elevation steppes and the wetter, higher elevation alpine grasslands (Doležal et al., 2024). We expected different traits to predict species' elevational optima in these contrasting habitats. Given the high phylogenetic diversity among the 310 plant species studied, belonging to 45 families and 171 genera, we also assessed whether the predictive power of functional traits remained significant after accounting for phylogenetic relationships by employing a species‐based trait–optima analysis with phylogenetic correction (Orme et al., 2013). Specifically, our research addresses the following questions. (1) How do morphological, anatomical, and physiological traits influence the elevational optima of plant species across the 3,500‐m gradient in Ladakh? (2) What are the differential impacts of moisture and cold limitations on trait–optima relationships in contrasting habitats (dry steppe vs. wetter, colder alpine)? (3) Which combinations of functional traits are most predictive of elevational optima in the studied plant species?

We hypothesized that plants with specific morphological traits (e.g., small plant size, root depth) will achieve higher elevational optima in regions with extreme cold and moisture limitations due to their adaptive advantages in resource utilization and stress resistance. Additionally, we expect that in wetter, colder alpine habitats, plant species will show stronger correlations between physiological traits (e.g., carbohydrate and nutrient storage) and elevational optima compared to species in dry steppe habitats, where morphological characteristics may be more significant due to water scarcity. Furthermore, we anticipate combining morphological, anatomical, and physiological traits will provide a more accurate predictive model for elevational optima than individual traits alone, indicating that plants employ multiple strategies to adapt to environmental gradients.

## RESULTS

### Trait differences between alpine and steppe species

Comparing the alpine and steppe species revealed that alpine plants are characterized by shorter height, slower growth (as indicated by narrower ring widths), and a higher proportion of storage tissues, which may reflect strategies to survive extreme environmental conditions (Table 1). They also exhibit greater root nutrient concentrations and elevated free sugar content, which may support survival under nutrient‐poor, colder conditions. In contrast, steppe plants tend to show greater height, faster growth, and higher mechanical tissue investment, likely as an adaptation to more competitive, resource‐rich environments.

On average, steppe plants are taller (42 cm) than alpine plants (20 cm) (*P* < 0.0001). Similarly, the mean ring width, which reflects annual growth, was also significantly reduced in the alpine environment (0.25 mm) compared to the steppe (0.45 mm) (*P* < 0.0001). The proportion of mechanical lignified tissue was significantly lower in the alpine (23%) than steppe (34%) species (*P* = 0.0025), indicating reduced structural reinforcement at higher elevations. Conversely, storage parenchymatic tissue was significantly higher in the alpine (63%) than in the steppe (49%) (*P* = 0.00068), reflecting a greater investment in resource storage. Maximum plant age (longevity) was higher in steppe than in alpine species (*P* = 0.0047). However, starch content and fructan content do not differ significantly between the environments. The free sugar content was significantly greater in the alpine than in the steppe (*P* = 0.004), likely serving as an adaptive mechanism to endure seasonal variability. For root nutrient content, RNC and RPC are both significantly higher in alpine (1.2% and 0.14%) than in steppe (1% and 0.12%) species. However, no significant differences are observed in LNC, LPC, or LCC between the environments. Differences in leaf δ^15^N isotope values are also highly significant, with a greater value in the steppe (*P* = 0.0003), indicating possible variations in nutrient‐use efficiency. Leaf δ^13^C isotope values, by contrast, do not exhibit significant differences between environments.

### Functional trait adaptations across elevational gradients

**Figure 1:**
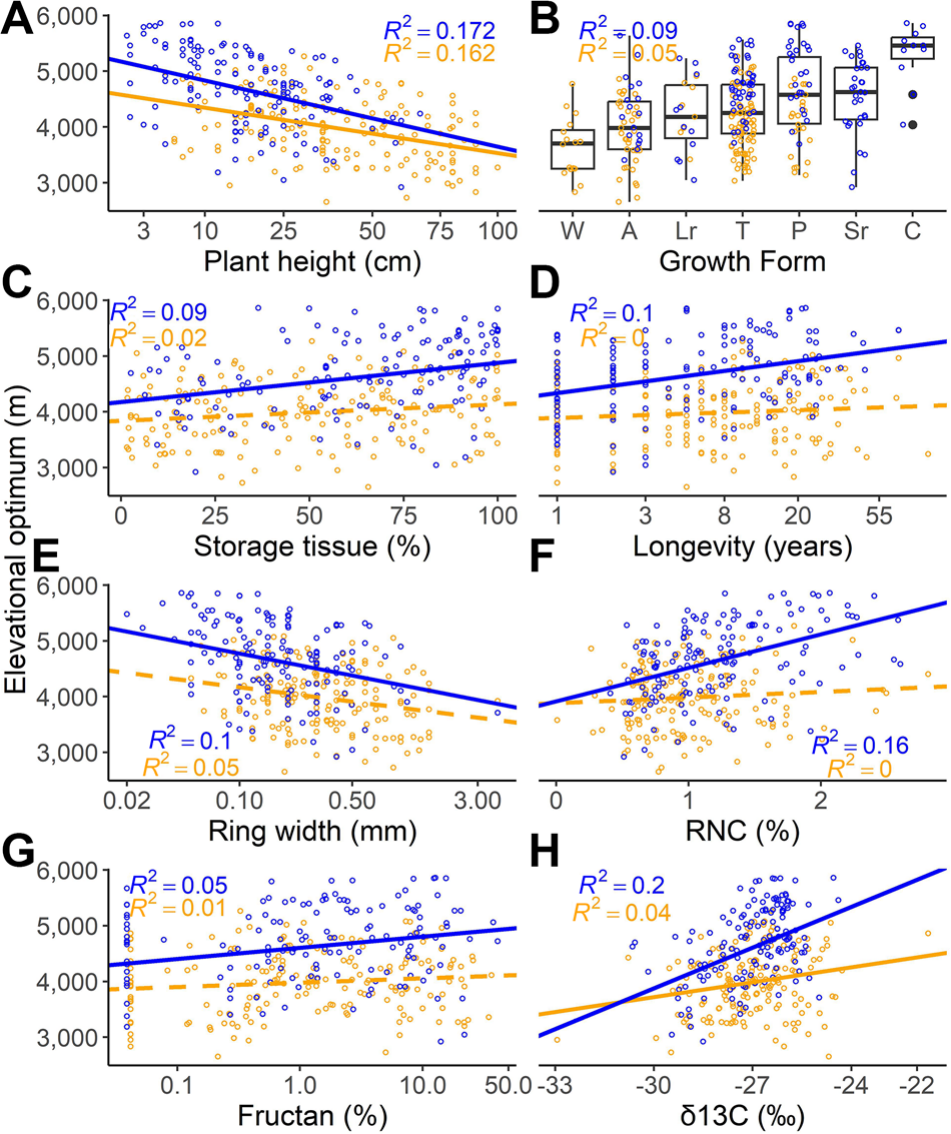
The relationships between functional traits and elevational optima (**A**–**H**) Blue points represent alpine species and orange points represent steppe species. The solid lines indicate selected predictors based on sequential approach utilizing phylogenetic linear models, while the dashed lines indicate non‐selected predictors. The growth forms are labeled as follows: A, annuals, C, cushions; Lr, clonal plants with long rhizomes; P, pleiocorms; Sr, clonal plants with short rhizomes; T, forbs with taproots; W, woody plants.

Alpine species demonstrated stronger and more frequent significant correlations between their traits and elevational optima compared to steppe species (Figure 1; Tables 1, S1), highlighting the essential role of functional traits in adapting to the more challenging conditions present at higher elevations. The PCA biplot illustrates the multidimensional relationships among traits, revealing trade‐offs between various anatomical and physiological traits of different organ and tissue types in relation to species distribution optima. The first two components account for 33.9% of the total variance among alpine species and 31.8% among steppe species.

**Figure 3:**
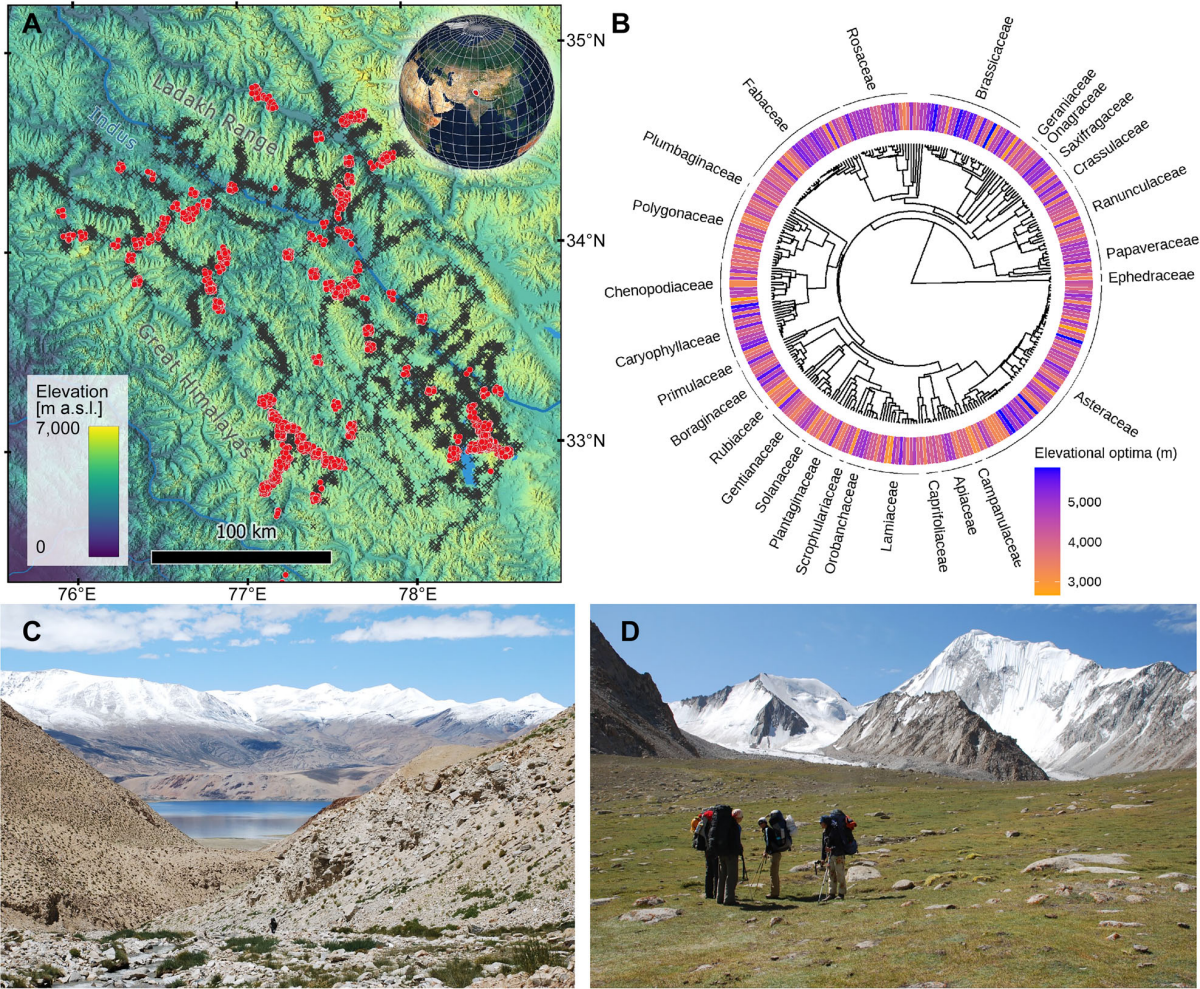
Study region, and phylogenetic tree of 310 vascular plant species studied with visualization of their elevational optima **(A)** Our study region in Ladakh, NW Himalayas, India. **(B)** Phylogenetic tree of 310 vascular plant species studied with visualization of their elevational optima. Ladakh has two contrasting environments across 2,650 to 6,150 m elevation gradients: dry steppes and semi‐deserts at lower elevations **(C)**, wetter and colder alpine and subnival zones at higher elevations **(D)**. Black crosses on the topographic map with elevation gradients mark 4,062 localities (each 100 × 100 m), where over 95,000 floristic records on species distributions were collected. Red dots indicate specific localities where plant trait data were gathered.

Single‐predictor analyses between plant elevational optima and functional traits revealed that plant height (Figure 1A), mean ring width (Figure 1E), and mechanical lignified tissue are associated with lower elevational optima. In both environments (steppe: *P* < 0.001; alpine: *P* < 0.001), taller plants are more likely to occur at lower elevations (Figure 1A), suggesting that species at these elevations invest in height, potentially competing for light in denser vegetation. Similarly, the mean ring width, which reflects annual growth, is greater at lower elevations (steppe: *P* = 0.002; alpine: *P* < 0.0001), indicating that species in less extreme conditions exhibit faster growth (Figure 3E). In the alpine environment, species with a higher proportion of mechanical lignified tissue are associated with lower elevation optima (*P* = 0.0006), suggesting that structural reinforcement is more advantageous in environments with less pronounced stresses, such as wind and snow loads.

In contrast, several traits exhibit positive relationships with elevational optima, indicating that species possessing these traits are more likely to occur at higher elevations. For instance, parenchymatic tissue is significantly higher in alpine species found at higher elevations (*P* = 0.0001, Figure 1C). This suggests that species at higher elevations prioritize resource storage to cope with shorter growing seasons and environmental stress. Similarly, longevity increases with elevation (*P* = 0.0001), particularly in the alpine species (Figure 1D). This implies that long‐lived species are better adapted to slower growth rates and harsher conditions at higher altitudes.

Nutrient‐related traits also show a positive correlation with elevational optima in the alpine environment (Figure 1F). Both RNC (*P* < 0.0001) and RPC (*P* = 0.001) increase with elevation, suggesting that species at higher elevations invest more in root nutrient acquisition to survive in nutrient‐poor soils. Additionally, leaf δ^13^C isotope values show a strong positive relationship with elevational optima in the alpine species (*P* < 0.0001, Figure 1H), indicating greater WUE at higher elevations, which may be an adaptation to reduced water availability. Fructan content, another indicator of resource storage, also increases with elevational optima in the alpine regions (*P* = 0.004, Figure 1G), reflecting the importance of carbohydrate reserves for species enduring cold temperatures and seasonal resource scarcity.

However, several traits do not exhibit significant relationships with elevational optima in either environment. These include leaf nitrogen and phosphorus (LNC, LPC), starch content, and free sugar content, suggesting that these traits are less influenced by elevation or that their variability may depend on other ecological or environmental factors. Overall, the relationships between functional traits and elevational optima reveal distinct patterns of adaptation. Species with optima at lower elevations are characterized by greater height, faster growth (as indicated by wider ring widths), and higher mechanical tissue investment, which may enhance their competitive ability in more favorable environments. In contrast, species with optima at higher elevations exhibit traits associated with resource conservation and stress tolerance, such as greater storage tissue, increased root nutrient content, enhanced WUE, and longer lifespans, all of which likely improve survival in the harsher, resource‐limited conditions at high altitudes.

### Integrating multiple traits to explain elevational distributions in Himalayan plants

#### Species' elevational optima are shaped by morphological traits

**Figure 2:**
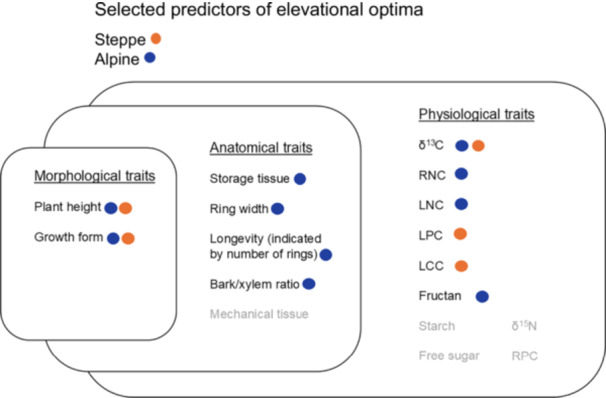
Examined functional traits, with orange points representing selected trait predictors of elevational optima for steppe species and blue points for alpine species Initially, we selected morphological traits, which were then used as covariates in selecting anatomical characteristics. Finally, physiological traits were added, utilizing the selected variables from the previous categories as covariates at each step.

Our hierarchical framework, which integrates morphological, anatomical, and physiological traits, revealed that the best‐performing model for steppe plants included both plant height and growth form as predictors, accounting for 19.89% of the variability in the data (adjusted *R* ^2^) (Figure 2). A model excluding plant height performed significantly worse (ΔAIC = 28.1), highlighting its strong influence. In contrast, omitting the growth form had a smaller impact on model performance (ΔAIC = 3.82). Elevational optima decreased as plant height increased. Species with high elevational optima included those with longer rhizomes (mean = 4,285 m n.m., *SD* = 513), forbs with pleiocorms (mean = 4,060 m n.m., *SD* = 559), and plants with taproots (mean = 4,045 m n.m., *SD* = 525). Conversely, species with lower optima tended to be annuals (mean = 3,856 m n.m., *SD* = 593) and woody shrubs (mean = 3,639 m n.m., *SD* = 542).

**Figure 4:**
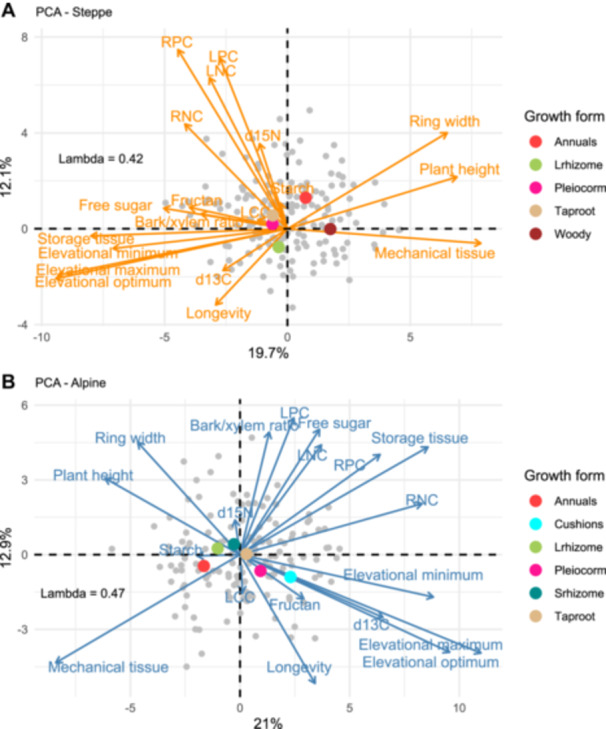
Multivariate phylogenetic principal component analysis (PCA) of plant traits and elevational distribution **(A**, **B)** Phylogenetic PCA for steppe **(A)** and alpine **(B)** species. Continuous plant traits, elevational optima, maxima, and minima were included in this analysis. Pagel's lambda and percentage of explained variability are shown in the plot. Plant growth forms were passively projected into the PCA bi‐plots: Annuals (monocarpic plants), Taproot (polycarpic non‐clonal forbs with main taproots), Pleiocorm (non‐clonal tap‐rooted herbs with long underground branches), Srhizome and Lrhizome (clonal plants with short or long rhizomes), Cushions (compact‐canopy alpine plants), Woody (shrubs and subshrubs).

Plant height and growth form were the primary predictors of elevational optima for alpine plants. This model explained a significantly greater variability in the data (adj. *R* ^2^ = 39.75%) compared to the model for steppe species, indicating a stronger influence. The model's fit declined markedly when plant height was omitted (ΔAIC = 35.39) or growth form was excluded (ΔAIC = 16.88). As with steppe species, elevational optima decreased with increasing plant height. Species with high elevational optima included cushion plants (mean = 5,298 m n.m., *SD* = 547) and forbs with pleiocorms (mean = 5,023 m n.m., *SD* = 635). In contrast, species with lower optima were primarily annuals (mean = 4,339 m n.m., *SD* = 612) and, unlike steppe species, those with long rhizomes (mean = 4,130 m n.m., *SD* = 742).

#### Anatomical traits enhance elevational optima predictions for alpine species

In the case of alpine species, incorporating anatomical traits significantly enhanced the model fit. The selected model included, in addition to covariates, the bark/xylem ratio, storage tissue, longevity, and ring width. This model accounted for 50.57% of the variability in the data (adj. *R* ^2^), representing an improvement of 10.82% in explained variability. Among the predictors, storage tissue was the only one that strongly influenced model fit when omitted (ΔAIC = 16.94). In contrast, the impacts of the other predictors were smaller: ring width (ΔAIC = 1.85), longevity (ΔAIC = 1.28), and bark/xylem ratio (ΔAIC = 0.87). Elevational optima increased with greater storage tissue and longevity, while they decreased with ring width and bark/xylem ratio. In contrast, the best‐performing model for steppe plants included only the covariates selected from the previous category. This suggests that no additional anatomical traits contributed to predicting the elevational optima of steppe species beyond plant height and growth forms.

#### Physiological traits as key predictors of elevational optima in steppe and alpine plants

From physiological traits, LPC, δ^13^C, and LCC were selected in steppe species, and explained variability increased to 25.67% (adj. *R* ^2^) (improvement by 5.78%) (Figure 1). The performance of the best‐fitting model was affected the most after omitting δ^13^C (ΔAIC = 8.11) while omitting LPC had a smaller effect (ΔAIC = 2.98), and omitting LCC barely affected the model fit (ΔAIC = 0.07). Elevational optima increased with LPC, δ^13^C, and LCC.

In alpine plants, LNC, RNC, δ^13^C, and fructan were selected as predictors from the final category and improved explained variability to 60.82% (adj. *R* ^2^) (by 10.25%). Model fit was considerably affected after omitting RNC (ΔAIC = 15.59), δ^13^C (ΔAIC = 9.4), and fructan (ΔAIC = 4.55), while omitting LNC (ΔAIC = 0.03) had almost no effect. Elevational optima decreased with LNC and increased with RNC, δ^13^C, and fructan.

In all selected models for both categories, the phylogenetic signal (*λ*) was 0 (*λ* = 0 corresponds to no phylogenetic signal, *λ* = 1 corresponds to a Brownian model of evolution).

## DISCUSSION

This study integrates morphological, anatomical, and physiological traits to predict the elevational optima of over 300 plant species distributed along a broad elevational gradient in the western Himalayas. As hypothesized, multiple traits from each category interact to determine species' elevational optima, underscoring the diverse adaptations required for survival along these gradients. This aligns with similar studies across large environmental ranges (Stahl et al., 2014; Midolo et al., 2019; Maharjan et al., 2021; Liu et al., 2022; Sigdel et al., 2023). By integrating traits across these three categories, we reveal the mechanistic underpinnings of species adaptations to varying environmental pressures. In particular, the predictive power of functional traits was more pronounced in the alpine species facing more extreme abiotic stress than the steppe species, where such selective pressures are less intense. Consistent with our hypothesis, high‐elevation species adopt conservative strategies to endure extreme abiotic stress. In contrast, steppe species rely on competitive and drought‐adaptive traits for resource‐limited but less severe environments, in accordance with previous studies (e.g., Liancourt et al., 2017). These findings enhance our understanding of how traits mediate species distribution and provide predictive insights into vegetation shifts under climate change.

### Elevational optima and functional trade‐offs

Our findings emphasize the critical role of functional trade‐offs in shaping species' elevational optima. These trade‐offs balance acquisitive and conservative strategies (Grime, 1974; Garnier et al., 2015), enabling plants to thrive across elevation gradients from 2,650 to 6,150 m, spanning dry steppes to cold alpine zones. Alpine species prioritize traits for survival under extreme conditions, while competitive and drought avoidance traits determine species distribution in steppes, consistent with previous studies (Dong et al., 2017). In alpine ecosystems, traits explain 60.82% of the variation in elevational optima, reflecting strong associations between traits and species success. This high explanatory power highlights the need for specialized traits to withstand low temperatures, high UV radiation, and resource scarcity (Dvorský et al., 2015; Chondol et al., 2024). Near the upper elevational limit for vascular plants (6,150 m), intense selective pressures drive the evolution of survival‐enhancing traits, such as efficient water and nutrient storage and compact growth forms (Klimešová et al., 2011; Angel et al., 2016; Dolezal et al., 2016). These traits are pivotal in defining alpine species' elevational optima under extreme conditions.

In the steppe zone at lower elevations, traits account for only 25.67% of the variation in elevational optima, reflecting less extreme but significant selective pressures. Steppe species face ecological factors like competition and water scarcity (Liancourt et al., 2017, 2020; Liancourt and Doležal, 2023) and often exhibit drought avoidance traits, such as deep roots and water conservation mechanisms (Schenk and Jackson, 2002). While these traits influence elevational optima, the selective pressures are less specialized than the alpine zone (Dong et al., 2017). This contrast highlights how functional traits evolve differently across elevation gradients to meet distinct environmental challenges.

### Morphological traits as key predictors

Morphological traits are among the most visible adaptations to elevation, as they influence plant size, form, and growth strategies (Dvorský et al., 2011). Plant height and growth form emerged as critical predictors of elevational optima in both steppe and alpine environments. Species with higher elevational optima tend to be smaller, a trend consistent with reduced biomass production under low temperatures and short growing seasons (Bliss, 1956; Halbritter et al., 2018). Smaller sizes at higher elevations help plants minimize exposure to freezing temperatures and strong winds while maximizing soil heat absorption (Jones, 1992; Neuner et al., 2000; Gardiner et al., 2016), which offers mechanistic advantages such as reduced freeze‐induced cavitation risk (Preston et al., 2006).

Our results also emphasize the role of growth form in climate decoupling. Compact growth forms, such as cushion plants, dominate the alpine environment and provide thermal advantages by creating warmer microclimates (Arroyo, 2003; Körner, 2021). Cushion plants dominate extreme subnival environments above 5,500 m (Dvorský et al., 2015) due to their heat‐retention properties (Sklenář et al., 2016; Rai et al., 2024). In contrast, long rhizomes, associated with lower elevation species, support nutrient acquisition and resilience against grazing (Doležal et al., 2024). Conversely, rhizome growth is limited in high‐elevation alpine terrains with poorly developed soils, favoring compact growth forms (Klimešová et al., 2011; Doležal et al., 2024). Perennial steppe plants with taproots, on the other hand, have higher elevation optima, likely due to their ability to survive in cold, dry soils (Klimešová et al., 2011). Annuals are more common at lower elevations, where drought is the primary limiting factor. Liancourt et al. (2020) suggested that plants in the dry steppes of the western Himalayas escape drought stress by timing their growth to favorable spring conditions.

### Anatomical traits in alpine adaptations

Anatomical traits significantly influenced elevational optima in alpine species but not steppe species, supporting our hypothesis that elevation‐specific adaptations are more crucial in extreme environments (Sperry, 2003; Ganthaler et al., 2022). High‐elevation alpine plants had more parenchyma storage tissue, an adaptation for enduring long dormancy periods and frost protection, which aligns with other similar studies (Hiltbrunner et al., 2021; Chlumská et al., 2022). Increased parenchyma tissue enhances carbohydrate storage (Dolezal et al., 2019a) and resilience to freeze–thaw cycles (Pastorczyk et al., 2014). Parenchyma cells contain high NSC. A higher proportion of carbohydrate‐accumulating parenchyma cells lowers osmotic potential and aids in repairing hydraulic disruptions such as freeze‐induced cavitation (Liu and Osborne, 2013; Trifilò et al., 2019). Our findings suggest that the proportion of storage tissue is a critical factor influencing species' elevational optima, a conclusion supported by the various advantages conferred by this adaptation.

Species with higher elevational optima tend to be long‐lived and exhibit small annual growth increments, similar to other studies (Chondol et al., 2023; Rai et al., 2024). Plants prioritize storage reserves and vegetative growth over reproduction in stable, resource‐poor environments to minimize mortality risks (Rosbakh and Poschlod, 2018). This slow, conservative growth strategy is well‐suited for high‐elevation habitats (Rosbakh and Poschlod, 2018; Schweingruber et al., 2020; Körner and Hiltbrunner, 2021). The persistence promoted by this life strategy also explains the small radial growth increments observed in species with higher elevational optima (von Arx et al., 2006). However, short growing seasons may also contribute to reduced ring width. Additionally, we found that species at high elevations had a lower bark‐to‐xylem ratio, possibly due to the high carbon content in the bark. We hypothesize that thinner bark helps balance the increased demand for carbon in the form of NSC, which alpine plants rely on for survival during extended dormancy and subsequent regrowth in the next growing season (Körner, 2021).

### Physiological traits and resource efficiency

Physiological traits such as nutrient content, WUE, and NSC concentration further explained elevational optima. Increased δ^13^C (used as a proxy for WUE) was associated with higher elevational optima in both alpine and steppe environments. Plants with increased WUE might better cope with temperature fluctuations and consequent freeze–thaw cycles (Dolezal et al., 2016). However, we note that Körner et al. (1991) proposed δ^13^C increase with elevation caused by changes in oxygen partial pressure and leaf thickness.

The relationship between elevation and nutrient content is a widely discussed topic. We discovered that steppe species with the higher elevation optima had elevated leaf phosphorus content, possibly to enhance photosynthetic activity (Kirschbaum and Tompkins, 1990) and, therefore, acquire more carbon during a short vegetation season (Körner, 2021). On the other hand, species with high elevational optima in alpine environments allocated a lot of nitrogen to their roots. Morecroft et al. (1992) proposed that slow‐growing plants do not incorporate as much carbon into their tissue, which results in high nutrient concentrations. However, our results show a strong relationship between RNC and elevation optima after controlling for ring width, which can serve as a proxy for plant growth. We thus argue that accumulating root nutrients might benefit high mountain plants. We hypothesize that high nitrogen concentration in roots could serve as a storage reserve, promoting growth and enhancing photosynthetic capacity after a dormant period.

We also found that steppe species with high‐elevation optima had greater leaf carbon content. Leaves with high carbon content are more resistant, which makes them less susceptible to stressful conditions (Wilson et al., 1999). On the other hand, alpine plants in the highest elevations did not show elevated LCC. We attribute this to the extremely short vegetation season, with little time to produce highly resistant tissue in leaves that only live one season. We also discovered that alpine species with high elevational optima had elevated fructan concentrations. Please note that our model already accounts for the effect of storage tissue fraction, which could be proportional to overall NSC storage (see Methods). Fructan is associated with freezing resistance (Livingston et al., 2009; Yoshida, 2021), which gives an undoubtable advantage to high mountain plants (Vijn and Smeekens, 1999).

### Methodological considerations and future directions

Given the vast geographic extent, rugged topography, and logistical constraints of the western Himalayas, the collection of distribution and trait data for over 300 plant species was carried out over multiple years. While this approach may raise concerns about data consistency, all sampling was carefully planned and conducted within the same phenological window each year to ensure temporal comparability and minimize seasonal bias, with the support of coordinated fieldwork and experienced personnel.

Our analytical emphasis on species' elevational optima was motivated by their utility in capturing the central tendencies of trait distribution along environmental gradients. A multivariate phylogenetic PCA of species' elevational optima, minima, and maxima in relation to functional traits revealed consistent patterns across these parameters. This finding supports our decision to prioritize elevational optima as a robust and ecologically meaningful metric that reflects where species are most successful and their core functional strategies. Nonetheless, elevational minima and maxima also carry valuable ecological information, particularly for understanding stress tolerance, range boundaries, and potential responses to climate change, and warrant further exploration in future studies.

We also acknowledge that plant trait expression can vary across years due to inter‐annual climatic fluctuations. Prior research by our team (e.g., Doležal et al., 2018) has demonstrated that growth patterns can shift under such variability, indicating a degree of trait plasticity. However, the relatively low intraspecific variation observed in our dataset supports the assumption that year‐to‐year variation is unlikely to alter the overarching elevational patterns reported here.

Lastly, our methodological and analytical decisions were shaped by both ecological objectives and practical constraints. By integrating traits from multiple functional categories and standardizing sampling protocols, we aimed to strike a balance between the depth of inference and data reliability. We encourage future research to build upon this framework by incorporating morphological, anatomical, and physiological traits to explain expanded parameters of species range distributions, together with experimental approaches that directly test trait plasticity and performance under changing environmental conditions.

## CONCLUSION

This study underscores the pivotal role of functional traits in determining species' elevational optima, highlighting their adaptations to abiotic and biotic pressures along steep environmental gradients. We identified distinct trait patterns associated with species' elevational optima in contrasting environments by examining 17 traits across over 300 species spanning elevations from 2,650 to 6,150 m. High‐elevation optima are best predicted by small stature, compact growth forms, enhanced storage tissues, and high WUE, which enable species persistence in extreme conditions. Species with high‐elevation optima adopt a conservative life strategy characterized by slow growth, longevity, and carbon allocation toward storage tissues rather than mechanical support. High‐elevation plants also exhibit elevated nutrient concentrations in their leaves and roots, as well as significant fructan storage, further supporting their adaptation to resource‐limited conditions. In contrast, lower elevation steppe and semi‐desert species are shaped by less extreme but still significant selective pressures, such as competition and water scarcity. Traits that promote competitive dominance and drought avoidance, such as deep roots and resource‐conserving strategies, play a more significant role in their distribution. These findings highlight the complexity of adaptations required for survival in extreme environments, indicating that further research on various plant traits is necessary to fully understand the relationship between species' elevational distribution and functional traits. This knowledge is vital for predicting vegetation shifts in response to climate change in mountain ecosystems.

## MATERIALS AND METHODS

### Study area

We collected plant distributional and trait data in Ladakh, located in the northwest Himalayas of India, covering an area of approximately 80,000 km^2^ with elevations ranging from 2,650 to 6,150 m a.s.l. (Figure 3A). The region encompasses various landscapes, with semi‐deserts and steppes prevailing in lower elevations (Figure 3C), while alpine grasslands predominate at higher elevations (Figure 3B; Dvorský et al., 2011). The vegetation shift is associated with a notable drop in mean annual temperatures along the elevational gradient, from 13°C at lower elevations to −13°C at the highest points, and an increase in precipitation from 50 to 250 mm (Dvorský et al., 2015; Macek et al., 2021). Seasonal temperature variations are marked by a difference between the average maximum temperature of the warmest month and the average minimum temperature of the coldest month, averaging 46°C ± 5°C across the gradient (Macek et al., 2021). Most precipitation falls as snow at higher elevations and sporadic rainfall at lower elevations (Dvorský et al., 2015).

### Elevational optima

We collected extensive field data on species elevational distribution across the Ladakh region, with floristic records ranging from 2,640 m a.s.l. in the Suru Region (northwest Ladakh) to 6,150 m a.s.l. in the Changthang Region (eastern Ladakh). Species distributions were based on 95,812 floristic records collected between 1997 and 2014 from 4,062 localities, each 100 × 100 m (Figure 3A). A total of 1,395 vascular plant taxa have been recorded. Of these, 310 species with comprehensive data on elevational distribution and functional traits were selected to analyze the relationships between traits and elevation optima. Species‐specific elevational optima, minima, and maxima were estimated using Huisman–Olff–Fresco (HOF) hierarchical regression models (Huisman et al., 1993; Dvorský et al., 2017). We determined species optima from the generated species response curves as the point of maximum species response, indicating the highest probability of occurrence. The elevational optima of the plant species varied from 2,652 to 5,862 m, encompassing species from two distinct habitats (Figure 3C, D): (1) dry steppe at lower elevations, with species optima ranging from 2,652 to 5,266 m; and (2) wetter, colder alpine zones at higher elevations, with species optima spanning from 2,921 to 5,862 m.

### Plant collection and trait measurement

To characterize trait differences among species, we collected over 7,800 individuals from 310 commonly occurring dicot herb and shrub species during our summer expeditions (July–August) in Ladakh from 2012 to 2017 (Figure 3A). Plant traits were measured over multiple years due to logistical constraints in sampling over 300 species across an 80,000 km^2^ area at extreme elevations. All samples were collected during the peak growing season using a standardized protocol to control temporal variation. Most sampled species were long‐lived perennials, with roots and rhizomes that can persist for up to 10–30 years (Chondol et al., 2023), ensuring that traits reflected long‐term growth and resource accumulation. We selected 20–30 undamaged individuals for each species, ensuring they were sampled at elevations corresponding to their elevational optima, as determined by maximum species density and abundance. This approach allowed us to capture trait expressions under conditions where species are best adapted and most competitive. We collected healthy, fully developed plants when they exhibited optimal growth during the growing season. This approach helped minimize variability related to developmental stages or environmental stressors.

After extracting the plants, we measured their height and carefully cleaned the roots to eliminate excess soil. The plants were then sorted into various organ types, including reproductive organs, leaves, stems, and belowground roots and rhizomes. To analyze NSC, we cut 3–5 cm segments from the coarse roots or rhizomes and immediately boiled them in 50% ethanol after harvest. This step was taken to prevent the rapid enzymatic hydrolysis of NSC (Dolezal et al., 2019b; Chlumská et al., 2022). The remaining plant parts were subsequently dried for further analysis of their traits in the laboratory. Specifically, leaf and root samples were prepared for nutrient and stable isotope analyses. This systematic approach enabled a thorough examination of the key functional traits of each organ.

### Functional trait measurements

We measured a suite of functional traits in at least 10 individuals per species to investigate how species' elevational optima relate to their ecophysiological adaptations. These traits included leaf carbon, nitrogen, and phosphorus concentrations (LCC, LNC, and LPC), as well as root nitrogen and phosphorus concentrations (RNC and RPC). These measurements allowed us to infer plant resource economics. Additionally, stable carbon isotope ratios (δ^13^C), which serve as proxies for WUE (Farquhar et al., 1989; Liancourt et al., 2020), and stable nitrogen isotope ratios (δ^15^N), indicative of nutrient acquisition and use efficiency (Robinson, 2001), were analyzed. Total carbon and nitrogen content, along with δ^13^C and δ^15^N, were measured at the Stable Isotope Facility at UC Davis, USA, using an elemental analyzer–isotope ratio mass spectrometer (EA–IRMS). Phosphorus concentrations were determined via spectrophotometry following HClO_4_ digestion using a SHIMADZU UV‐1650PC spectrophotometer.

Non‐structural carbohydrate concentrations in belowground samples, including starch, fructans, and various free sugars, were assessed using ethanol extraction, centrifugation, and filtration, followed by ion‐exchange chromatography (Chlumská et al., 2022). The starch content was determined using the Megazyme total starch assay procedure (www.megazyme.com), and ethanol‐soluble sugars were quantified through anion exchange chromatography with pulsed amperometric detection. Soluble sugars included sugar alcohols such as glycerol, xylitol, and arabitol, and simple sugars such as glucose, fructose, sucrose, and galactose (for details, see Chlumská et al., 2022).

#### Microscopic analysis of growth rings in root collars

The semi‐arid conditions and seasonal climate variability of the western Himalayas have driven perennial plant species to develop deep root systems with distinct ring‐porous or semi‐ring‐porous annual growth rings. These features allow precise estimation of plant age and radial growth rates (Doležal et al., 2018; Chondol et al., 2023). To determine plant age and radial growth history for herb and shrub plants collected for leaf and root trait analyses, we obtained approximately 2 cm segments from the root collar zone, the transition between root and stem, which represents the oldest part of the plant (Schweingruber et al., 2005; Gärtner and Schweingruber, 2013). These segments were immersed in a 50% aqueous ethanol solution to preserve tissue flexibility and prevent mold growth, before anatomical analysis of plant age and radial growth histories. We prepared cross‐sections of root collars in the anatomical laboratory using a sled microtome. We stained them with a 1:1 mixture of Astra Blue and Safranin to differentiate lignified (fibers, vessel secondary cell walls) and nonlignified (living cells, axial and radial parenchyma) tissues (see Doležal et al., 2018 for details). Astra Blue stained parenchyma cells blue, while Safranin stained lignified cells pink (Gärtner and Schweingruber, 2013). These sections were mounted with Canada Balsam, and high‐resolution microscopic images were captured to count annual growth rings and measure their width along two radii for the oldest individuals of each species (Chondol et al., 2023). The average ring width, calculated from measurements along two radii, was used to estimate the annual growth rate per individual, which was then averaged across individuals to determine species‐specific growth rates.

#### Quantitative tissue analysis: insights into plant structure and resource allocation

In addition to growth rates, we analyzed double‐stained cross‐sections to quantify the proportion of three primary tissue types: parenchymatic (storage), water‐conductive (transport), and mechanical (support). These tissues play critical roles in plant survival under harsh conditions. Using ImageJ software, we measured the relative areas of conduits (white), fibers (red‐stained), and parenchyma (blue‐stained) by randomly drawing 100 polygons that covered a quarter of the stem's cross‐sectional area (Dolezal et al., 2019a). Furthermore, we calculated the bark‐to‐xylem ratio to evaluate resource allocation strategies between protective tissue (bark) and mechanical tissue (xylem) (Rosell et al., 2017).

#### Categorization of plant growth forms

We categorized each species into seven morphological groups based on their structural characteristics and ecological strategies to explore the relationship between plant growth forms and their elevational preferences. These groups included monocarpic plants, polycarpic non‐clonal forbs with taproots, non‐clonal tap‐rooted herbs (pleiocorms), clonal plants with short or long rhizomes, compact alpine cushion plants, and woody shrubs (Klimešová et al., 2011). Monocarpic plants, which are typically short‐lived, are often associated with disturbed vegetation at lower elevations. Polycarpic non‐clonal forbs with taproots are commonly found in semi‐deserts and steppes, environments usually characterized by water scarcity. Pleiocorms, possessing small, deep roots connected by underground branches, are adapted to rocky alpine habitats such as subnival zones and screes. Clonal plants with either short or long rhizomes exhibit different spreading rates associated with environments ranging from saline wetlands to alpine grasslands. Compact alpine cushion plants dominate higher elevations, creating dense structures that trap heat and form favorable microclimates. Woody shrubs, on the other hand, are prevalent in lower elevation steppes.

### Species phylogeny

To evaluate the potential influence of phylogenetic relatedness on the relationship between species traits and elevational optima, we reconstructed the phylogeny of the studied species using nucleotide sequences from GenBank (www.ncbi.nlm.nih.gov/nuccore/). We employed a combined multigene approach, achieving maximum data density with four loci: the internal transcribed spacer (ITS), the trnT–trnL intergenic spacer, the matK + trnK region, and the rbcL gene. Sequence alignment was performed using the L‐INS‐i algorithm in the online version of MAFFT 6 (http://mafft.cbrc.jp/alignment/server). Phylogenetic analysis was conducted using Bayesian inference in MrBayes 3.1.2, with the resulting tree (Figure 3B) serving as the basis for phylogenetic linear models in the trait–environment analysis.

### Data analysis

We first conducted a multivariate phylogenetic principal component analysis (PCA) to examine the relationships among plant traits and species distribution optima, minima, and maxima, analyzing species separately within steppe (169 taxa) and alpine (141 taxa) habitats (Figure 4). This analysis revealed consistent trends of optima, minima, and maxima, supporting our decision to focus on species elevational optima in relation to the morphological, anatomical, and physiological traits sampled at their corresponding elevations.

Furthermore, we employed phylogenetic linear models to assess the individual impact of plant functional traits on species elevational optima, analyzing species separately in steppe and alpine habitats. The predictors were categorized into three groups: (1) morphology (including plant height and growth form); (2) anatomy (comprising bark/xylem ratio, longevity indicated by the number of rings, mechanical tissue, storage tissue, and annual growth rates indicated by average ring width); and (3) physiology (encompassing traits such as LNC, LPC, RNC, RPC, stable isotopes δ^15^N and δ^13^C, LCC, and reserve storage compounds like fructan, starch, and free sugars).

Initially, we selected predictors from the morphology category, which were then used as covariates for selecting predictors from the anatomy category. These were subsequently combined as covariates for the physiology category to evaluate which functional traits from the latter groups improved the model fit. This approach enabled us to assess the effects of functional traits while taking into account the interconnections between morphology, anatomy, and physiology.

To identify relevant predictors, we explored all possible combinations of predictors (including models with no predictors or only covariates) for each category using the Akaike Information Criterion (AIC) and selected the model with the best performance. Additionally, we considered models with similar fit (ΔAIC < 2) to identify traits that may have been omitted from the selected model due to their strong correlation with other predictors. However, if a model demonstrated a better fit after excluding a predictor, we did not consider that predictor as influencing elevational optima. The importance of each predictor in the model was determined by the change in model fit (ΔAIC) when that predictor was omitted.

The sequential approach outlined above allowed us to leverage our prior expectations regarding the interrelationships among functional traits, thereby minimizing potential biases associated with analyzing many correlated traits. However, we also present a more straightforward perspective through single‐predictor relationships between each functional trait and elevational optima (Tables 1, S1). We tested the significance of these models and employed the Holm–Bonferroni method to control the family‐wise error rate. We applied square root or natural logarithm transformations to address skewed distributions among predictors. To enable logarithmic transformation of variables containing zeros, we substituted them with half of the second smallest value. We quantified the phylogenetic signal using the maximum likelihood estimation of Pagel's lambda for each model. All analyses were conducted using R version 4.3.2, utilizing the caper package (Orme et al., 2013) to fit phylogenetic models and the phylolm package (Ho et al., 2016) to assign missing species to the phylogenetic tree, and the phytools package (Revell, 2012) to assess the trait interrelationships using phylogenetic PCA.

**Table 1:** A list of plant traits studied in steppe and alpine species

Functional trait	Abbreviation	Steppe vs. alpine (*P*‐value)	Steppe	Steppe (*P*‐value)	Alpine	Alpine (*P*‐value)
Plant height	Height	< 0.0001*	42 (5–100)	< 0.0001 (↓*)	20 (2–67)	< 0.0001 (↓*)
Growth form	—	—	—	0.02 (n.s.)	—	0.003 (*)
Bark/xylem ratio	—	< 0.0001*	0.59 (0.03–5)	0.57 (n.s.)	1.1 (0.1–5)	0.36 (n.s.)
Mechanical lignified tissue (%)	Mechanical tissue	0.0025*	34 (0–95)	0.053 (n.s.)	23 (0–87)	0.0006 (↓*)
Storage parenchymal tissue (%)	Storage tissue	0.00068*	49 (1.5–100)	0.04 (↑n.s.)	63 (3–100)	0.0001 (↑*)
Maximum plant age (years)	Longevity	0.0047*	12 (1–101)	0.22 (n.s.)	10 (1–70)	0.0001 (↑*)
Mean ring width (mm)	Ring width	< 0.0001*	0.45 (0.05–4)	0.002 (↓*)	0.25 (0.02–4)	< 0.0001 (↓*)
Leaf nitrogen content (%)	LNC	0.052	2.4 (0.6–5.6)	0.26 (n.s.)	2.6 (1.2–7.3)	0.54 (n.s.)
Leaf phosphorus content (%)	LPC	0.4	0.18 (0.06–0.7)	0.13 (n.s.)	0.18 (0.08–0.6)	0.80 (n.s.)
Leaf carbon content (%)	LCC	0.09	40 (25–47)	0.3 (n.s.)	40 (30–47)	0.996 (n.s.)
Root nitrogen content (%)	RNC	< 0.0001*	1 (0.01–2.8)	0.29 (n.s.)	1.2 (0.4–2.6)	< 0.0001 (↑*)
Root phosphorus content (%)	RPC	< 0.0001*	0.12 (0.02–0.37)	0.24 (n.s.)	0.14 (0.04–0.3)	0.001 (↑*)
Leaf δ^13^C isotope (‰)	δ^13^C	0.037	−27 (−33 to −22)	0.004 (↑n.s.)	−27 (−31 to −24)	< 0.0001 (↑*)
Leaf δ^15^N isotope (‰)	δ^15^N	0.0003*	3.4 (−2 to 16)	0.31 (n.s.)	1.8 (−4 to 16)	0.78 (n.s.)
Starch content (%)	Starch	0.01	4 (0–30)	0.78 (n.s.)	7 (0–47)	0.22 (n.s.)
Fructan content (%)	Fructan	0.48	4 (0–36)	0.13 (n.s.)	5 (0–41)	0.004 (↑*)
Free sugar content (%)	Free sugar	0.004*	4.7 (0.2–20)	0.54 (n.s.)	5.9 (0.5–36)	0.60 (n.s.)

Table includes mean values, ranges, and statistical tests for differences between the two categories, as well as single‐predictor relationships between plant elevational optima and functional traits. Upward arrows (↑) and downward arrows (↓) indicate positive and negative relationships, respectively. An asterisk denotes a significant relationship, while “n.s.” indicates a non‐significant relationship. We utilized the Holm–Bonferroni method to control for the family‐wise error rate.

## CONFLICTS OF INTEREST

The authors declare no conflicts of interest.

## AUTHOR CONTRIBUTIONS

J.B. and J.D. conceptualized the study, J.B. performed the statistical analysis and wrote the original manuscript. J.D. acquired the funding and curated the data, and M.M. provided modeled elevational optima. All authors reviewed and edited the manuscript.

## Supporting information

Additional Supporting Information may be found online in the supporting information tab for this article: http://onlinelibrary.wiley.com/doi/10.1111/jipb.13971/suppinfo

**Table S1.** Explained variability and phylogenetic signal (Pagel's lambda) in predictors of elevational optima.

Supporting information Appendix

## Data Availability

Data are available in Supporting Information Appendix.
