# Supplementary material for: Integrating morphological, anatomical, and physiological traits to explain elevational distributions in Himalayan steppe and alpine plants
Source: J Integr Plant Biol. 2025 Jul 15;67(10):2643–57. doi: 10.1111/jipb.13971 (PMC12498082; doi:10.1111/jipb.13971)
Supplement: Supplementary file 1 — Table S1. Explained variability and phylogenetic signal (Pagel's lambda) in predictors of elevational optima. [file JIPB-67-2643-s001.docx]

| **Functional trait** | **Steppe** | | **Alpine** | |
| --- | --- | --- | --- | --- |
|  | **Adj. R^2^** | **Lambda** | **Adj. R^2^** | **Lambda** |
| Plant height | 0,162 | 0 | 0,172 | 0,4 |
| Growth form | 0,05 | 0 | 0,09 | 0,27 |
| Bark/Xylem ratio | 0 | 0 | 0 | 0,43 |
| Mechanical tissue | 0,02 | 0 | 0,07 | 0,41 |
| Storage tissue | 0,02 | 0 | 0,09 | 0,419 |
| Longevity | 0 | 0 | 0,1 | 0,4 |
| Ring width | 0,05 | 0 | 0,1 | 0,42 |
| LNC | 0 | 0 | 0 | 0,43 |
| LPC | 0,01 | 0 | 0 | 0,44 |
| LCC | 0 | 0 | 0 | 0,44 |
| RNC | 0 | 0 | 0,16 | 0,39 |
| RPC | 0 | 0 | 0,07 | 0,45 |
| δ13C | 0,04 | 0 | 0,2 | 0,39 |
| δ15N | 0 | 0 | 0 | 0,43 |
| Starch | 0 | 0 | 0 | 0,42 |
| Fructan | 0,01 | 0 | 0,05 | 0,45 |
| Free sugar | 0 | 0 | 0 | 0,45 |

**Table S1.** Explained variability and phylogenetic signal (Pagels lambda) in predictors of elevational optima. Parameters are derived from models with predictors transformed as described in Methods.
